# Generation of Thyroid Follicular Cells from Pluripotent Stem Cells: Potential for Regenerative Medicine

**DOI:** 10.3389/fendo.2014.00096

**Published:** 2014-06-19

**Authors:** Will Sewell, Reigh-Yi Lin

**Affiliations:** ^1^Department of Otolaryngology – Head and Neck Surgery, Saint Louis University School of Medicine, St. Louis, MO, USA

**Keywords:** thyroid, thyroid follicular cells, stem cells, embryonic stem cells, pluripotent stem cells, induced pluripotent stem cells, regenerative medicine, cell replacement therapy

## Abstract

Nearly 12% of the population in the United States will be afflicted with a thyroid related disorder during their lifetime. Common treatment approaches are tailored to the specific disorder and include surgery, radioactive iodine ablation, antithyroid drugs, thyroid hormone replacement, external beam radiation, and chemotherapy. Regenerative medicine endeavors to combat disease by replacing or regenerating damaged, diseased, or dysfunctional body parts. A series of achievements in pluripotent stem cell research have transformed regenerative medicine in many ways by demonstrating “repair” of a number of body parts in mice, of which, the thyroid has now been inducted into this special group. Seminal work in pluripotent cells, namely embryonic stem cells and induced pluripotent stem cells, have made possible their path to becoming key tools and biological building blocks for cell-based regenerative medicine to combat the gamut of human diseases, including those affecting the thyroid.

## Introduction

The thyroid gland synthesizes and releases two essential hormones into the bloodstream. These hormones impact virtually every tissue and cell in the body and play a role in regulating a wide variety of biological processes, such as metabolism, brain development, body temperature, weight, and much more. Thyroid disease is fairly common with more than 12% of the population experiencing a thyroid related condition at some point during their lifetime in the United States alone (http://www.thyroid.org/thyroid-events-education-media/about-hypothyroidism/). Thyroid disorders manifest in a variety of ways, which include: hypothyroidism, hyperthyroidism, thyroid nodules, thyroiditis, thyroid cancer, and goiters. Each disorder has a number of potential underlying causes. Untreated, a number of serious health conditions, such as cardiovascular disease and osteoporosis may result from thyroid disease. Even though nearly all thyroid diseases are life-long afflictions, the good news is, with proper medical attention they may be managed over the patient’s lifetime.

For a decade now, researchers have been exploring stem cell technologies for the treatment of thyroid disorders (1–6). Successful cell repair/replacement would eliminate a patient’s life-long dependence on synthetic thyroid hormone replacement. At the heart of this cell-based regenerative medicine reside pluripotent stem cells (PSCs). PSCs are a special group of stem cell defined by a few essential, yet distinguishing characteristics; (1) the ability to self-renew, (2) prolonged undifferentiated proliferation with little or no karyotypic abnormalities, and (3) the potential to differentiate into every cell lineage/type in the body. Recently, the future of thyroid disease treatment took a giant step forward when transplanted mouse embryonic stem cell (mESC) derived thyroid follicles fostered symptomatic recovery in athyroidic mice (4).

## Derivation of ES Cells

Embryonic stem cells (ESCs) originate from the inner cell mass (ICM) of blastocyst-stage embryos (7–9). Originally, mESC lines were established by two similar, yet distinct, approaches. Evans and Kaufman employed ovariectomy and hormonal stimulation of pregnant female mice to impair implantation and induce developmental delay of embryos (7). Collected and cultured ICMs developed into egg cylinder-like bodies that were picked, dissociated, and passaged onto fibroblast feeders. Alternatively, Gail Martin harvested early embryos from mated, superovulated female mice (8). The trophectoderm was removed by immuno-surgery, resulting in isolation of the ICM. It would not be until the late 1990s that the first human ESC lines would be created after cleavage stage embryos produced by *in vitro* fertilization were cultured to the blastocyst stage (9). Original methods to culture ESCs simplified the isolation of pluripotent cells by circumventing the need to “convert” an embryo into a tumor *in vivo*. What’s more, it made possible the isolation of ESCs from both non-inbred and mutant mouse strains. Finally, these studies provided evidence of a teratocarcinoma-derived growth factor essential for stimulating non-differentiating proliferation of mESCs from cultured ICMs.

## Maintaining ES Cells *in vitro*

Once established, ESCs must be cultured under conditions that promote undifferentiating proliferation. For the most part, general media components, such as a feeder cells, Dulbecco’s Modified Eagle medium, and fetal bovine serum (FBS) are essentially the same for standard culturing of virtually all ESCs. However, species-specific ingredient requirements for non-differentiating growth do exist. Differentiation-inhibiting activity (DIA, the key cytokine secreted by feeders) was shown to be closely related structurally and functionally to leukemia inhibitory factor (LIF) (10). Exogenous, recombinant LIF is capable of substituting for DIA, thereby eliminating the need for feeders in mESC culturing.

The conditions for long-term culturing of human ESCs, however, differ from mouse in two fundamental ways. First, LIF plays no apparent role in undifferentiated proliferation (11). Secondly, exogenous bFGF is required when hESCs are cultured long-term under serum-free conditions (12). However, feeder-free culturing on a matrix (matrigel or laminin) is capable of supporting undifferentiated proliferation (13). Recently, hESC culturing was transformed when a feeder-free culturing system demonstrated prolonged cell culturing without loss of either pluripotency or differentiation potential, or acquisition of karyotypic abnormalities, even at clonal dilutions (14, 15).

When pluripotency promoting conditions are removed, ESCs will differentiate. Under suitable conditions, cell lineages deriving from the embryonic endoderm, mesoderm, and ectoderm will be produced (16). Assessment of the differentiation potential of ESCs is essential for their usefulness in research and future therapeutic applications. Transplanting cells into immunodeficient mice results in growth of a differentiated tumor (teratoma) composed of all three germ layers. Alternatively, *in vitro* differentiation may be initiated by allowing fully or partially dissociated ESCs to cluster together under non-attachment conditions to form spherical colonies, known as embryoid bodies (EBs) (16, 17). EBs may be further differentiated by prolonged culturing following attachment to a substrate. *In vitro* differentiation of ESCs into a broad spectrum of lineages has been tremendously advanced by development of many new suitable culture conditions and protocols. This type of differentiation system has the potential of functioning as an unlimited source of cells for cell-based regenerative medicine and the treatment of a wide-range of diseases, including thyroid disorders.

## Thyroid Ontogeny and Differentiation of Thyroid Follicular Cells from ES Cells

The thyroid gland is composed of two kinds of endocrine cells: thyroid follicular cells (TFC) and parafollicular C cells. TFCs, which make up the bulk of the gland, are derived from foregut endoderm and produce/secrete thyroid hormones T_3_ and T_4_. Parafollicular C cells on the other hand, derive from the neuroectoderm and synthesize calcitonin. During embryogenesis, the anteroposterior axis of the foregut endoderm is laid out into organ-specific domains that include the primordial thyroid, lung, pancreas, and liver (18, 19). Expression of defined sets of transcription factors at distinct locations along the anteroposterior axis of the definitive endoderm (DE) derived primitive gut demonstrates the first sign of regional specification of organ domains (19). During early-to-mid embryogenesis, a small group of cells destined to become TFCs are found in the primitive pharynx and are set apart by their co-expression of *Nkx2-1*, *Foxe*, *Pax8*, and *Hhex*. Each of these genes is expressed in other tissues outside of the thyroid; however, their co-expression is restricted solely to the thyroid, where they direct thyroid organogenesis by establishing a thyroid-specific gene expression program (20). TFCs are further characterized by their exclusive expression of thyroglobulin (*Tg*) and thyroperoxidase (*TPO*), as well as thyroid stimulating hormone receptor (*TSHR*) and sodium/iodide symporter (*NIS*), which display extrathyroidal expression (20). Evidence suggests that *Nkx2-1* and *Pax8* are mostly responsible for driving thyroid-specific activation of *Tg*, *TPO*, and *NIS* genes (21, 22). Despite its lack of specificity, thyroid agenesis in *Nkx2-1* knockout mice and pediatric hypothyroidism in humans born with *NKX2-1* mutations further highlight the importance of *Nkx2-1* as a key regulator of thyroid organogenesis (23, 24). Similarly, *Pax8* knockout mice exhibit a total lack of TFCs and some cases of congenital hypothyroidism in humans are found to be associated with *PAX8* mutations (25, 26).

*In vitro* differentiation of mESCs into TFCs was first demonstrated by our laboratory in 2003 by the expression of thyroid lineage markers in cultured EBs (1). It was also shown that continued *Pax8* and *TSHR* expression in EB-derived monolayers required TSH under serum-free conditions, whereas *Tg* expression was lost. A few years later, TFCs arranged in follicle-like clusters were derived from an enriched population of ESCs expressing a *TSHR* driven GFP when cultured on a basement membrane matrix in the presence of TSH following insulin and insulin-like growth factor-1 stimulation (2, 6). In order to more efficiently specify TFCs *in vitro*, cells require methodically guided culturing conditions conducive to initiate DE formation followed by anterior foregut endoderm (AFE) induction. Although an efficient inducer of mesoderm derived lineages; FBS is a poor endoderm inducer (27). One noteworthy observation however was that restricting mESC exposure to serum enhances DE associated gene expression (27). Based on developmental findings in *Xenopus*, supplementing medium with activin effectively promotes DE specification in both mouse and human ESCs (27). Yet, DE produced from activin induction alone withstands thyroid lineage specification, thus requiring additional culturing modifications. NOGGIN (BMP signaling antagonist) and SB-431542 (pharmacological inhibitor of activin A/nodal and TGF-β signaling) emerged from a study testing the ability of various morphogens and inhibitors to push DE into AFE (28). Furthermore, addition of a growth factor cocktail composed of WNT3α, KGF, FGF10, BMP4, and EGF further guided hESCs toward a ventral AFE fate (28). However, supplementation of this cocktail with FGF2 was necessary for efficient induction of *Nkx2-1* in mESCs (29). Ultimately, these findings suggest that thyroid competent cells (evidenced by *Nkx2-1*, *Foxa2*, and *Pax8* expression) are effectively generated in endoderm progenitors by stage-specific and time-dependent BMP/TGF-β inhibition followed by combinatorial induction of BMP and FGF signaling (29). Thus far, directed development of TFCs has only been substantiated with mESCs and is yet uncertain whether current protocols and methodologies would have similar developmental consequences on hESCs.

**Figure 1:**
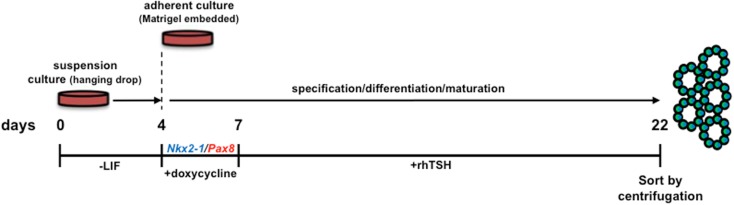
**Schematic diagram of the protocol used for *in vitro* differentiation of thyroid follicles from a tetracycline-inducible murine *Nkx2-1-Pax8* ESC line**. LIF, leukemia inhibitory factor; rhTSH, recombinant human thyroid stimulating hormone. Initial culturing of ESCs in hanging drops (without LIF supplementation) allows for embryoid body differentiation. *Nkx2-1* and *Pax8* are temporally induced by doxycycline, thereby directing differentiation toward a thyrocyte fate. Forced expression of *Nkx2-1/Pax8* results in robust thyroid stimulating hormone receptor expression, suggesting cells are capable of responding to rhTSH. Therefore, the sequential treatment of embryoid bodies with Dox-rhTSH dramatically enhances thyrocyte lineage specification and the formation of follicle-like structures, which are subsequently isolated for transplantation [Ref. (4)].

*Nkx2-1* and *Pax8* were shown to synergize in activating expression from thyroid-specific promoter/enhancer driven constructs in a hepatic cell line, however, endogenous thyroid-specific gene expression was not detected (21). Two studies recently revealed that *Nkx2-1* and *Pax8* overexpression in mESCs can direct differentiation into TFCs *in vitro* and organize into three-dimensional neo-follicles following TSH treatment (4, 5) without the need for enrichment of lineage-directed cells using cell sorted fluorescent reporter knock-in ESC lines (2). Remarkably, transient *Nkx2-1*/*Pax8* overexpression was capable of inducing follicle-like formation *in vitro* without early activin induced endoderm specification (Figure 1) (4). This approach obviates the complex, laborious culture requirements needed to direct differentiation of “unmodified” ESCs. What remains to be seen is whether transient, insertion-less strategies to ectopically express *Nkx2-1*/*Pax8* with subsequent TSH administration would be capable of eliciting the same outcome. Perhaps thyroid lineage cells may be enriched by utilizing fluorescence activated cell sorting to capture lineage directed, unmodified ESCs by interrogating cultured cells for expression of both NIS and TSHR.

## Induced Pluripotent Stem Cells

**Figure 2:**
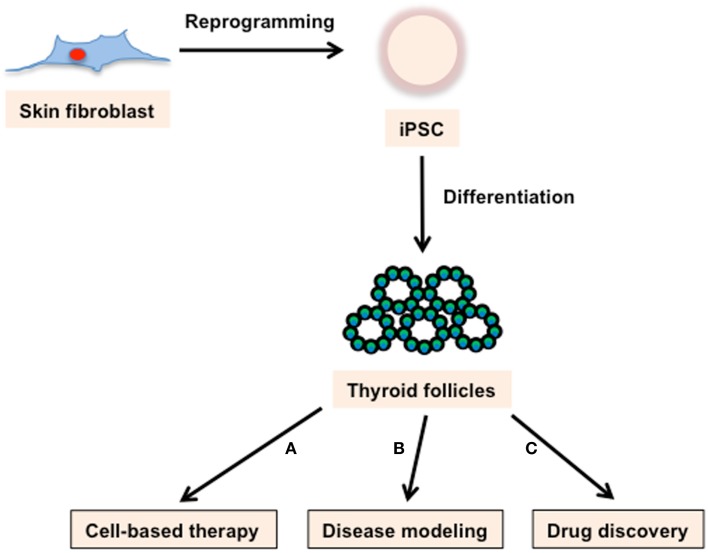
**Applications of iPSC technology**. Skin fibroblast-derived iPSCs can produce thyroid follicles. These cells can be used for **(A)** cell-based therapy, **(B)** disease modeling, and **(C)** drug discovery. **(A)** Based on the demonstrated success of ESCs in rescuing thyroid ablated mice, iPSCs derived thyroid follicles have incredible potential as a cell replacement therapy for a variety of thyroid maladies. **(B)** iPSC lines with genotypes characteristic of varied genetically based thyroid diseases could provide an avenue to study disease mechanisms as well as a screening system for evaluating pharmaceutical and recombinant approaches aimed at treating those diseases. **(C)** iPSCs derived thyroid follicles would enable easy quantification of drug effects on follicle structure and function.

In 2006, the milestone discovery that fibroblasts could be converted to PSCs, simply by forced expression of four transcription factors (*Oct4*, *Sox2*, *Klf4*, and *c-Myc*; collectively termed OSKM or Yamanaka factors), took the research community by storm (30). These iPSCs have broad applications, including: autologous cell-based therapy; the modeling of genetic disorders; the study of genetic variability; and functioning as a substrate for a variety of targeted therapeutic screens (Figure 2). In addition to transforming development and disease related research, iPSC technology tore down technical and ethical barriers that inevitably mired ESC-based regenerative medicine.

The Yamanaka factors reprogram somatic cells into ESC-like cells by (1) silencing somatic and retroviral genes, (2) activating a network of pluripotency genes, and (3) inducing epigenetic changes. When successfully reprogramed, iPSCs grow in tightly packed, domed colonies, and resemble ESCs in morphology and at the molecular and phenotypic levels; possess the ability to differentiate into derivatives of the three embryonic germ layers; and generate germline-competent chimeras. Table 1 shows the comparison between ESC and iPSC. The full developmental potential of miPSCs was shown when reprogramed cells produced full-term mice after injection into tetraploid blastocysts (31). Since its inception, iPSC production has gone through a tremendous number of metamorphoses that have exploited and modified several reprograming variables that include: donor cell type, reprograming cocktail, culture conditions, and integrative or non-integrative delivery systems. Different cell types reprogram with different efficiencies and kinetics. For instance, reprograming primary human keratinocytes by OSKM transduction is 100 times more efficient and twice as fast when compared to fibroblasts (32). Neural progenitor cells represent another example because they are capable of being reprogramed with only *Oct4* and *Klf4*, due to their endogenous expression of *Sox2* (33).

**Table 1:** **Comparison between ESC and iPSC**.

	iPS	ES cells
Reported in humans	Yes	Yes
Embryos or donor oocytes required	No	Yes
Stemness marker expressed	Yes	Yes
Teratomas produced	Yes	Yes
Utility as a research tool	Allows repeated development	Allows the study of development
Can be used as models for human diseases	Yes	Some
Can be used in a screen to identify drugs	Yes	Yes
Variable fates	Likely	Yes
Develop into specific human tissues	To be shown	Yes
Genetically match the patient	Unknown	No
Additional information	Cells are genetically modified in current methods	Cells are allogeneic and might cause immune rejection

*Adapted from Nishikawa et al. (34). With permission from Macmillan Publishers Ltd*.

Modifying or supplementing the core OSKM with other factors or small molecules (35, 36) influencing pluripotency, proliferation, and epigenetic remodeling have been shown to enhance the quality and efficiency of reprograming, in both mouse and human iPSCs, as well as promote pluripotency maintenance during culturing [reviewed in Ref. (37)]. Indeed, treatment of both mouse and human fibroblasts with small-molecule chemicals in combination with genetic factors dramatically improved reprograming efficiency and enabled reprograming with fewer genetic factors (35, 38). Recently, it was discovered that ectopic expression of the *miR302/367* cluster, combined with Hdac2 suppression, can directly and efficiently reprogram both mouse and human fibroblasts without supplementation with any of the Yamanaka factors (39).

Traditional retroviral delivery of reprograming factors admittedly introduces an element of risk of tumorigenesis from transplanted iPSC-derived cells due to reactivation of transgenes and/or insertional mutagenesis, thereby making them unsuitable for therapeutic applications in humans. For this reason, researchers have developed a variety of insertion-less approaches such as non-integrating viruses, plasmids, small molecules, transposons, recombinant proteins, episomal vectors, and modified RNAs to generate iPSCs [reviewed in Ref. (40)]. Despite their lure, many of these strategies are accompanied by limitations that cast a shadow of doubt about their therapeutic safety. Nonetheless, these and future approaches continue to bring the field of regenerative medicine closer to safe, efficient iPSC generation for patient treatment.

## Application of Pluripotent Stem Cells for Regenerative Medicine

Human ESCs and iPSCs have placed application of cell-based regenerative medicine for the treatment of a host of diseases, such as diabetes, Parkinson’s, and a variety of thyroid maladies, within reach. Today, the thyroid has now joined the list of body parts now “repairable” in mice. Costagliola’s group rescued mice afflicted with experimentally induced hypothyroidism by transplanting ESC-derived thyroid follicles (4). Ultimately, the challenge is to translate these findings into viable treatment strategies for humans.

Embryonic stem cells are ethically controversial because human embryos are destroyed for their derivation. Furthermore, concerns exist about immune rejection of ESC-derived tissues following transplantation into patients (30). Creation of genetically matched ESCs through therapeutic cloning would make it possible to circumvent graft versus host disease (GvHD). However, use of therapeutic cloning is still tainted by the destruction of an embryo, requires substantial banking of donated oocytes, is technically challenging, and is extremely inefficient. Oocytes derived from lineage-directed PSCs represent a possible remedy to scarce oocyte availability (41, 42). An alternative patient-specific cell source for treatment of thyroid disease comes from the discovery of a resident thyroid stem cell population (43–45).

Unlike ESCs, iPSCs are common and readily available, non-controversial, capable of being produced from a patient’s cells in a large number of academic and commercial laboratories, and represent virtually an endless supply of replacement tissues for the treatment of trauma as well as a broad spectrum of diseases. Regardless of the reprograming approach, before autologous cells can be used to regenerate/replace tissue lost or surgically removed due to disease, any underlying disease-causing genetic mutation(s) must be known. Ideally, donor cells would be interrogated for the presence of the genomic aberrations and then corrected after reprograming. Idiopathic thyroid diseases suspected of being caused by genetic or epigenetic defects can be surveyed for abnormalities with a variety of methods [reviewed in Ref. (46)] and innovations in genome editing, such as transcription activator-like effector nucleases, will significantly advance targeted gene alteration in human stem cells for therapeutic applications (47).

Before the practical application of PSCs in cell replacement therapy can be realized, a number of issues need to be addressed. The following talking points are by no means an exhaustive. (1) Establishment of xenogenic-free and chemically defined culturing conditions to reduce culture variability and eliminate the potential introduction of xenoantigens that elicit an immune response. (2) Protocols for efficient induction of the appropriate lineage. (3) Determination of maturation stage and the therapeutic cell dosage needed. (4) Stringent strategies for lineage selection and/or purging of undifferentiated cells to reduce PSC contamination of the graft, which can end in teratoma growth. (5) Donor/recipient compatibility resulting in GvHD. (6) Do the established differences between iPSCs and ESCs, such as epigenetics (aberrant epigenetic memory as well as memory of the donor cell) and mutational load translate into different therapeutic outcomes? (7) Development of techniques for administering the therapeutic cells into target location is vital to successful treatment.

## Conclusion

Diseases of the thyroid represent not only the most common endocrine disorder but the most common autoimmune disease (e.g., Hashimoto’s disease and Graves’ disease) as well (48). One considerable obstacle standing in the way of successful application of regenerative medicine approaches to treat many thyroid disorders is an underlying autoimmunity like patients exhibiting autoimmune thyroiditis type diseases. More than likely, cell replacement would be doomed unless the underlying autoimmunity is eliminated or suppressed. Cell-based immunotherapies targeting a variety of malignancies are now in clinical practice. Assorted immune system lineages, such as dendritic, T, and natural killer (NK) cells have been expanded *in vitro* then infused back into patients (49–51). A recent immunotherapy study using antigen-loaded dendritic cells yielded encouraging results in patients diagnosed with medullary thyroid carcinoma as evidenced by disease stabilization and immunological response in select patients (52). To augment this approach to combating neoplasias, hESC derived NK cells were shown to facilitate effective tumor clearance *in vivo* (53). This finding suggests that hESCs, and hiPSCs for that matter, are feasible cell sources for cancer immunotherapy.

In summary, PSCs hold incredible curative and therapeutic potential. Given the tremendous evolution in stem cell research and advances in lineage-directed culturing, especially those inducing TFCs, it is only a matter of time before cell-based regenerative medicine becomes a treatment option for some thyroid disorders.

## Conflict of Interest Statement

The authors declare that the research was conducted in the absence of any commercial or financial relationships that could be construed as a potential conflict of interest.
